# First record of leaf-hole shelters used and modified by leaf beetles (Coleoptera, Chrysomelidae), with descriptions of two new *Orthaltica* Crotch species from southern India

**DOI:** 10.3897/zookeys.336.5435

**Published:** 2013-09-27

**Authors:** Kaniyarikkal Divakaran Prathapan, Alexander S. Konstantinov, K. M. Shameem, A. P. Balan

**Affiliations:** 1 Department of Entomology, Kerala Agricultural University, Vellayani P.O., Trivandrum-695 522, Kerala, INDIA; 2 Systematic Entomology Laboratory, ARS, USDA, c/o National Museum of Natural History, Smithsonian Institution P.O. Box 37012, MRC-168, Washington, DC 20013-7012, U.S.A.

**Keywords:** Leaf-hole shelter, faeces, leaf beetles, new species, host plant

## Abstract

Behavioural novelties observed in adult leaf beetles of two new *Orthaltica* Crotch species include: 1) the use of low cost leaf-hole shelters, either in pre-formed holes produced by larger beetles that fed on the same leaf, or artificially created holes as part of an experiment; and 2) the use of faeces to partition the hole. Two new southern Indian species of the genus *Orthaltica* are described and illustrated: *Orthaltica syzygium* and *Orthaltica terminalia*. Host plants are identified for both species. A key to the Indian species of *Orthaltica* is provided.

## Introduction

Animal architecture reflects the biology of its builder. Three broad categories of animal constructions have been recognized: homes, traps and displays (Hansell 2005; 2007). Homes or shelters protect their builders from external physical and possible biological hostilities. They provide protection by means of their architecture, effecting avoidance of detection and the prevention of invasion after detection has occurred (Hansell 2005).

In architecture birds, spiders, termites, ants, bees and wasps are prodigies. Beetles exhibit the most numerous radiation within the animalia, but there are very few architects amongst them. In leaf beetles (Chrysomelidae) (about 50, 000 species) larval defensive structures, built of faeces and exuvial skin, are known (Chaboo et al. 2007, Chaboo et al. 2008, Prathapan and Chaboo 2011). Larvae of several leaf beetles are known to use faeces for defense, and nearly 20% of the described chrysomelid species have a form of faecal covering or faeces-associated structure at some point in their lifecycle (Vencl et al. 1999; Weiss 2006).

Two genera in the Cassidinae, namely *Imatidium* Fabricius (Gilbert et al. 2001) and *Leptispa* Baly (Maulik 1919; Voronova and Zaitsev 1982; Prathapan et al. 2009), have the only leaf beetles known to build leaf shelters. Larvae of nearly all of about 4500 species of Camptosomata (Cryptocephalinae and Lamprosomatinae) build cases from their own faeces (Chaboo et al. 2008).

**Figures 1–8.**
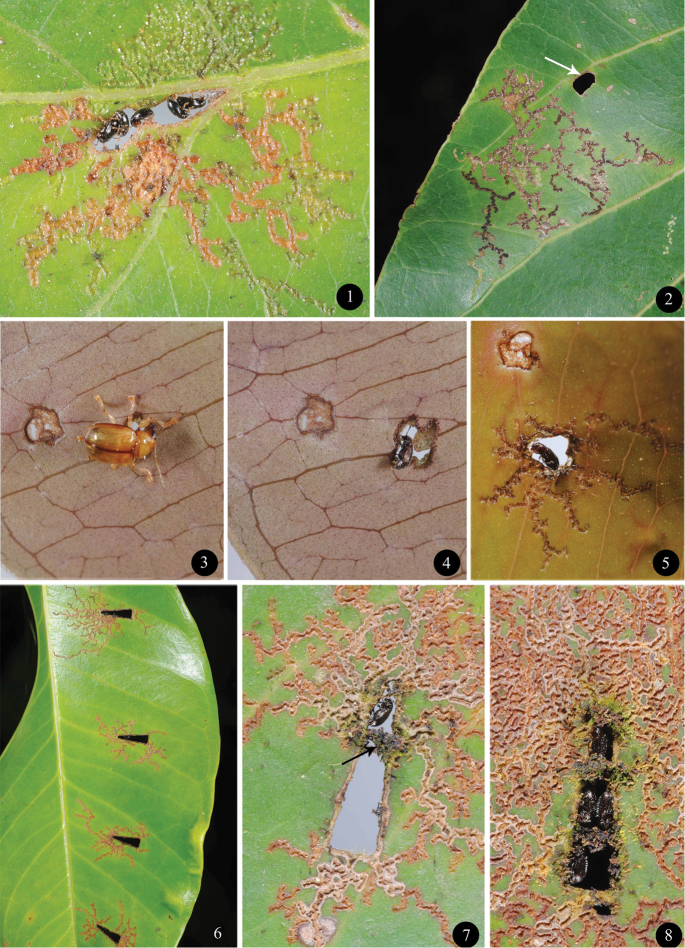
*Orthaltica* species and their low cost leaf-hole shelters. **1** Leaf-hole shelter of *Orthaltica terminalia* on *Terminalia paniculata*. Note the feeding trenches radiating from the leaf-hole shelter **2** Feeding trenches of non-shelter forming species on an unidentified plant from the Combretaceae. Note the unoccupied leaf-hole nearby **3** Eumolpine beetle (*Basilepta* sp.) feeding holes on the abaxial side of the leaf of *Syzygium caryophyllatum* **4** *Orthaltica syzygium* occupying the leaf-hole made by the eumolpine beetle in Fig. 3 **5** Leaf-hole shelter of *Orthaltica* in Fig. 3 as seen on the second day, viewed from adaxial side of leaf. Note the feeding trenches radiating from the leaf-hole **6** Triangular-shaped artificial leaf-holes on *Syzygium travancoricum* used as shelter by *Orthaltica syzygium* - note feeding trenches radiating from holes **7** Single occupancy - note the first partition **8** Multiple occupancy with multiple partitions.

Builders invest considerable resources and time making constructions. Hence, natural selection favours building behaviour which reduces the cost of building, whilst maximizing the benefits it offers. Nesting in existing cavities produced by primary cavity nesters, as in woodpeckers, is a common method of cost reduction used by birds. Low cost shelters of *Leptispa pygmaea* Baly larvae are formed by feeding alone—no cutting, bending or secretion of silk or glue is required (Prathapan et al. 2009). A type of low cost shelter, named a leaf-hole shelter, is here reported for the first time for two species of *Orthaltica* Crotch, a genus of minute leaf beetles (Figs 1, 4–8).

The genus *Orthaltica* is distributed in the Afrotropical, Australian, Nearctic and Oriental Regions (Seeno and Wilcox 1982; Reid 1990) and contains 44 named species, of which the majority (32 species) are distributed in the Oriental Region. The taxonomy of *Orthaltica* is complex, currently including eight generic synonyms. *Crioceris copalina* (F.), the type species, occurs in the Nearctic Region. In India the genus is represented by at least ten species level taxa, of which only six have been formally named and described: *Orthaltica assamensis* (Scherer, 1971), *Orthaltica bengalensis* (Basu & Sengupta, 1978), *Orthaltica coomani* (Laboissiére, 1933 in Basu and Sengupta 1978), *Orthaltica dakshina* (Basu & Sengupta, 1978), *Orthaltica minuta indica* (Medvedev, 1998) and *Orthaltica purba* (Basu & Sengupta, 1985). We here describe two species that utilise leaf-hole shelters. However, a revisionary study of the genus is necessary, at least in Asia, to clarify both species boundaries and the relationships between species groups currently placed in *Orthaltica*.

## Material and methods

Five species of *Orthaltica* were collected from various localities in southern India, along with their host plants, forming part of our long term study of the leaf beetles of India. Species from this genus are known to feed on plants from the Combretaceae, Melastomataceae and Myrtaceae. Leaf-hole sheltering, described below, was observed in two species feeding on *Syzygium* (Myrtaceae) and *Terminalia* (Combretaceae) species respectively. The *Orthaltica* species feeding on *Syzygium* (Gaertn.) is here described as *Orthaltica syzygium* new species, and the species on *Terminalia* L. as *Orthaltica terminalia* new species. *Orthaltica syzygium* was collected from various localities in the states of Karnataka (Kottigehara, Kudremukha) and Kerala (Arippa, Kumarakom, Vattavada) on *Syzygium cumini* (L.) Skeels, *Syzygium caryophyllatum* Alston and *Syzygium travancoricum* Gamble. *Orthalitca terminalia* was also collected in the states of Karnataka (Kudremukha) and Kerala (Calicut University Campus, Kallar, Trichur), but on *Terminalia cuneata* Roth and *Terminalia paniculata* Roth. The leaf-hole shelters on *Terminalia paniculata* were initially formed by a *Tricliona* sp. (Eumolpinae). Natural populations of all the *Orthaltica* species were carefully observed in the field for feeding and shelter seeking behaviour. Leaf-hole shelters of *Orthaltica syzygium* were observed at the Kudremukha National Park on *Syzygium caryophyllatum*, but were initially formed by a *Basilepta* sp. (Eumolpinae) (Fig. 3). Other leaf feeding beetles, but particularly Eumolpinae, were observed for their feeding activity on the hosts of *Orthaltica*. Artificial leaf holes were presented to the beetles on leaf laminae of *Syzygium travancoricum*. Holes were made with a punch, generally used for making the card points on which small leaf beetles are mounted. The punch created elongate, triangular holes of 7 mm in length and a width of 2 mm at the base. About 60 leaves were provided to adults of *Orthaltica syzygium* on 20^th^ April, 2013, each with six holes punched on the lamina. Occupation of the holes by these beetles was observed on 20^th^ May, 2013 and 20^th^ August, 2013 and could easily be determined by checking for the presence or absence of feeding trenches radiating from the hole (Fig. 6).

Descriptive terminology follows Konstantinov (1998). Holotypes of the new species are deposited in the National Museum of Natural History, Smithsonian Institution, Washington, DC, USA (USNM). Paratypes will be deposited in the National Pusa Collection, Indian Agricultural Research Institute, New Delhi, India (NPC); University of Agricultural Sciences, Bangalore, India (UASB); Natural History Museum, London, United Kingdom (BMNH); and in the personal collection of the first author (PKDC). Plant vouchers with accession numbers are deposited in the Calicut University Herbarium, Calicut, India. Different labels on specimens are denoted by numbers and separated by “;”.

## Results

### Orthaltica syzygium

Prathapan & Konstantinov sp. n.

http://zoobank.org/B1CFE822-AA5E-498C-8000-F64A0A5791F2

http://species-id.net/wiki/Orthaltica_syzygium

Figs 4 –15

#### Description.

**Figures 9–15.**
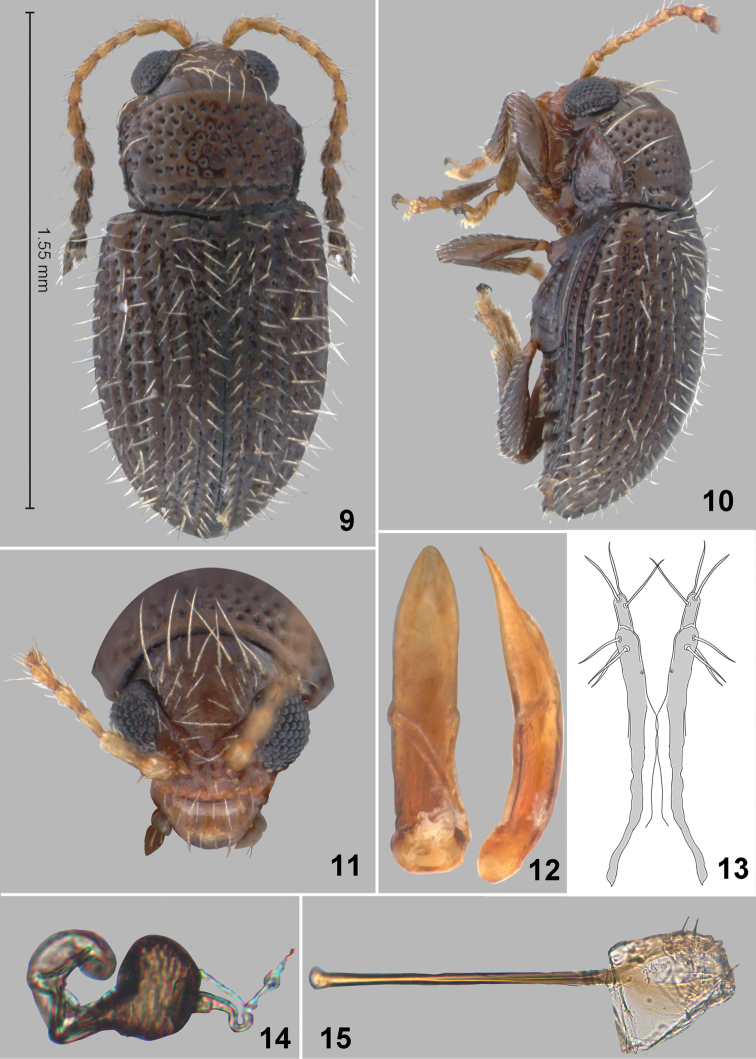
*Orthaltica syzygium* Prathapan & Konstantinov, sp. n. **9** Dorsal habitus **10** Lateral habitus **11** Head frontal view **12** Aedeagus, ventral and lateral view **13** Vaginal palpi **14** Spermatheca **15** Tignum.

Body length 1.30–1.60 mm, width 0.67–0.81 mm. Body dark brown (Figs 9, 10). Basal antennomeres and tarsi paler and yellowish-brown. Vertex with 8 long and more than 10 short setae. Supracallinal sulci straight; orbital sulci short, poorly developed; and suprafrontal and suprantennal sulci poorly developed. Antennal calli narrow and elongate (Fig. 11), relatively widely connected, with two large setiferous pores. Frontal ridge parallel-sided, ending before reaching anterofrontal ridge. Anterofrontal ridge of uniform height, but sloping abruptly towards clypeus. Labrum as wide as distance between outer edges of antennal sockets. Setiferous pores on dorsal surface of labrum small, not possible to count. Antennomere 3 slightly shorter than 4 and 5 separately.

Pronotum widening towards apex and narrowing abruptly after reaching anterolateral callosity. Lateral margins slightly uneven with a prominent submedial denticle, and two long setae (Fig. 10). Antebasal transverse impression distinct (Fig. 9). Pronotal surface densely covered with large punctures, their diameter larger than the distance between punctures.

Elytra densely and evenly pubescent (Figs 9, 10), with erect setae on interstices. Elytral punctures arranged in striae located in relatively deep grooves. Humeral calli distinct. Pro- and middle tibiae lacking spurs. Claws appendiculate.

Apex of aedeagus narrowing gradually (Fig. 12), base above basal opening as wide as medial width, and apex slightly down-curved in lateral view.

Spermatheca with short thick pump (Fig. 14), distinctly separated from receptacle. Tignum straight, widening abruptly anteriorly (Fig. 15). Apical abdominal segment attached near middle of vaginal palpi (Fig. 13).

#### Type material.

**Holotype**, male. 1) India: Kerala, Arippa, 8°50'11"N, 77°1'46"E, 30.xi.2011, 236m. D. Prathapan & K. Shameem Coll.; 2) H:396 *Syzygium travancoricum*; 3) *Orthaltica* sp. 1 KD Prathapan det 2012; 4) Holotype *Orthaltica syzygium* n. sp. Prathapan & Konstantinov 2012 (USNM). **Paratypes**: 13 specimens, the same labels as holotype; 8 specimens, the same labels as holotype except for the date 23.iii.2012. 29 specimens. 1) India Kerala, Kumarakom, 2.vii.2010 Prathapan Coll.; 2) 157 *Syzygium caryophyllatum*; 3) Paratype *Orthaltica syzygium* n. sp. Prathapan & Konstantinov 2012. 16 specimens. 1) India Karnataka, Kottigehara, 13°7'7.7"N, 75°30'37.9"E, 8.v.2011, 938m. D. Prathapan & K. Shameen Coll. 2) Host *Syzygium cumini* 3) Paratype *Orthaltica syzygium* n. sp. Prathapan & Konstantinov 2012. 19 specimens. 1) India Karnataka, Kudremukha N. P., 13°14'42"N, 75°6'44"E, 12.v.2011, 110m. D. Prathapan & K. Shameem Coll. *Syzygium caryophyllatum*; 2) Paratype *Orthaltica syzygium* n. sp. Prathapan & Konstantinov 2012 (5 – BMNH, 64 – NPC, 5 – PKDC, 5 – UASB, 9 – USNM).

#### Etymology.

The specific epithet is a noun in apposition, based on the host plant name.

#### Host plants.

*Syzygium cumini* (L.) Skeels (Accession no. 6516), *Syzygium caryophyllatum* Alston (Accession no. 6626) and *Syzygium travancoricum* Gamble (Accession no. 6693) (Myrtaceae).

#### Remarks.

*Orthaltica syzygium* can easily be distinguished from all known Indian *Orthaltica* species using the key provided below. *Orthaltica terminalia* can be distinguished from the most similar species using the following characters: body dark brown (Fig. 9); vertex with eight long and more than 10 short setae (Fig. 11); elytra densely pubescent (Figs 9–10); apex of aedeagus narrowing gradually (Fig. 12); and spermatheca with short thick pump (Fig. 14).

### Orthaltica terminalia

Prathapan & Konstantinov sp. n.

http://zoobank.org/040BDB2D-61C5-4E47-83DF-E15A898C06CC

http://species-id.net/wiki/Orthaltica_terminalia

Figs 1 , 16–22

#### Description.

**Figures 16–22.**
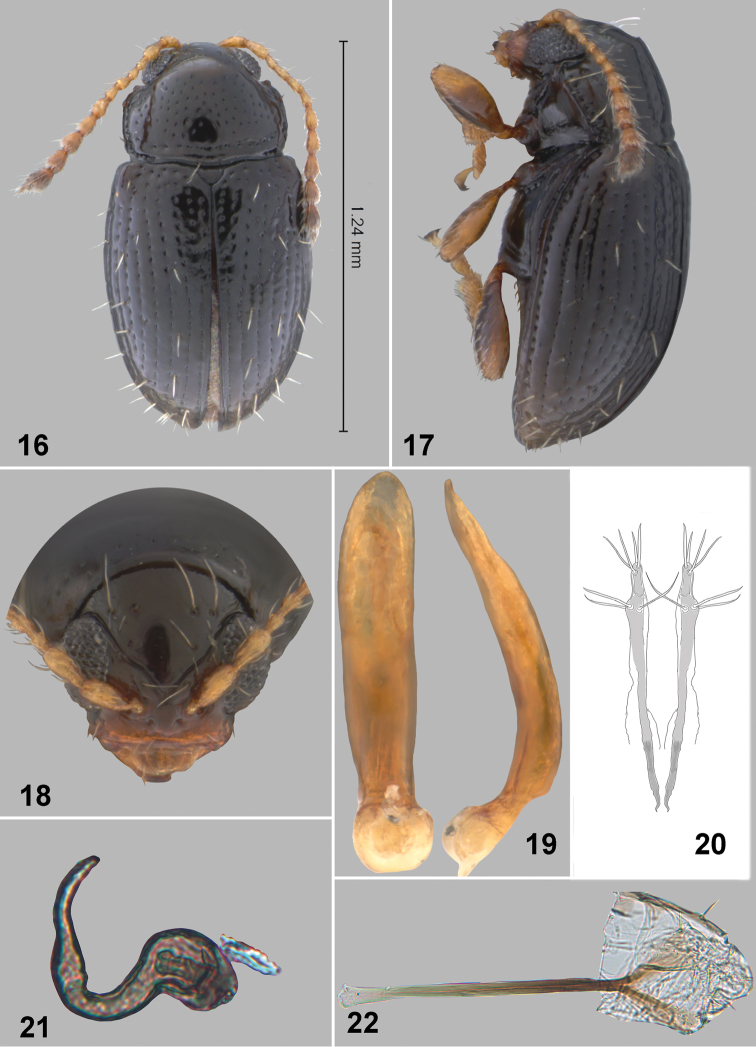
*Orthaltica terminalia* Prathapan & Konstantinov, sp. n. **16** Dorsal habitus **17** Lateral habitus **18** Head frontal view **19** Aedeagus, ventral and lateral view **20** Vaginal palpi **21** Spermatheca **22** Tignum.

Body length 1.20 – 1.50 mm, width 0.75 – 0.84 mm. Body shiny brownish-black (Figs 16, 17). Antennomeres (except most apical) and legs (except base of some femora) yellowish-brown. Vertex with 4 long and 6 short setae. Supracallinal sulci slightly curved; orbital sulci absent; suprafrontal sulcus poorly developed; and suprantennal sulcus shallow. Antennal calli narrow and elongate (Fig. 18), relatively widely connected, with two large setiferous pores. Frontal ridge parallel-sided, ending before reaching anterofrontal ridge. Anterofrontal ridge of uniform height, but sloping abruptly towards clypeus. Labrum as wide as distance between outer edges of antennal sockets. Setiferous pores on dorsal surface of labrum small, more than two, but not possible to count. Antennomere 3 slightly shorter than 4 and 5 separately.

Pronotum about as wide basally as apically, with sides evenly curved. Lateral margins slightly uneven and lacking a prominent denticle; bearing two long setae (Fig. 17). Antebasal transverse impression distinct (Fig. 16). Pronotal surface sparsely covered with slightly elongate punctures, their diameter smaller than distance between punctures.

Elytra sparsely pubescent (Figs 16, 17), with erect setae mainly near apices. Elytral punctures arranged in striae located in relatively deep grooves. Humeral calli distinct. Pro- and middle tibiae lacking spurs. Claws appendiculate.

Apex of aedeagus narrowing abruptly (Fig. 19), base above basal opening narrower than medial width, and apex slightly up-curved in lateral view.

Spermatheca with long thin pump (Fig. 21) which is not distinctly separated from receptacle. Tignum straight widening gradually anteriorly (Fig. 22). Apical abdominal segment attached to basal third of vaginal palpus (Fig. 20).

#### Type material.

**Holotype**, male. 1) India Karnataka, Kudremukha N. P., 13°14'42"N, 75°6'44"E, 12.v.2011, 110m. D. Prathapan & K. Shameem Coll. *Terminalia paniculata*; 2) Holotype *Orthaltica terminalia* n. sp. Prathapan & Konstantinov 2012 (USNM). **Paratypes**: 45 specimens, the same labels as holotype. 1 specimen 1) India Karnataka, Guddayanadoddi, 12°43.233'N, 077°33.576'E, 29.vii.2011 905m; 2) *Terminalia arjuna*; 3) *Orthaltica* sp. 2 K D Prathapan det. 2012; 4) Paratype *Orthaltica terminalia* n. sp. Prathapan & Konstantinov 2012. 12 specimens 1) India Kerala, Calicut Univ. Campus, 3.vii.2012, Shameem K. Coll. Ex *Terminalia paniculata*; 2) Paratype *Orthaltica terminalia* n. sp. Prathapan & Konstantinov 2012. 8 specimens 1) India Kerala, Trichur VellaniPacha, 27.x.2010, Prathapan K. D. Coll; 2) 230 *Terminalia arjuna*; 3) *Orthaltica* sp. 2 K D Prathapan det. 2012; 4) Paratype *Orthaltica terminalia* n. sp. Prathapan & Konstantinov 2012. 1 specimen 1) India Kerala, Aathirappilly, 10°18'44.7"N, 076°42'19.6"E, 26.x.2010, 570m; 3) *Orthaltica* sp. 2 K D Prathapan det. 2012;4) Paratype *Orthaltica terminalia* n. sp. Prathapan & Konstantinov 2012. 9 specimens 1) India Kerala, Kallar, 17.viii.2010, Prathapan Coll.; 2) *Terminalia paniculata*; 3) *Orthaltica* sp. 2 K D Prathapan det. 2012; 4) Paratype *Orthaltica terminalia* n. sp. Prathapan & Konstantinov 2012. (5 – BMNH, 53 – NPC, 5 – PKDC, 5 – UASB, 9 – USNM).

#### Etymology.

The specific epithet is a noun in apposition, based on the host plant name.

#### Host plants.

*Terminalia cuneata* Roth (Accession no. 6576) and *Terminalia paniculata* Roth (Accession no. 6484) (Combretaceae).

#### Remarks.

*Orthaltica terminalia* can be easily distinguished from all known Indian *Orthaltica* species using the key below. *Orthaltica syzygium* can be distinguished from the most similar species using the following characters: body shiny black (Fig. 16); vertex with four long and six short setae (Fig. 18); elytra sparsely pubescent (Figs 16 & 17); apex of aedeagus narrowing abruptly (Fig. 19); spermatheca with thin elongate pump (Fig. 21). *Orthaltica minuta indica* (Medevedev) is not included in the key as there is no character to separate it from *Orthaltica dakshina* (Basu & Sengupta). Both species are from the same locality (Doddabetta in Tamil Nadu) and further research may prove them to be synonymous.

##### Key to the Indian species of *Orthaltica*

**Table:** 

1	Length 2.4 mm; vertex with a pair of long widely separated setae at posterior	*Orthaltica purba* Basu & Sengupta
–	Length 1.6 mm or less; vertex with a row of at least four long setae at posterior	2
2(1)	Elytral interstices, between the eighth and ninth row of punctures, sharply carinated; transverse antebasal impression on pronotum sinuate	*Orthaltica assamensis* (Scherer)
–	Elytral interstices, between eighth and ninth row of punctures, not carinate; transverse antebasal impression on pronotum straight	3
3(2)	Anterofrontal ridge low, merging gradually with clypeus	*Orthaltica bengalensis* (Basu & Sengupta)
–	Anterofrontal ridge high; sloping abruptly towards clypeus	4
4(3)	General body colour reddish-brown	*Orthaltica dakshina* (Basu & Sengupta)
–	General body colour dark brown to black	5
5(4)	Frontal ridge merging gradually with anterofrontal ridge, together forming a triangular ridge	*Orthaltica coomani* (Laboissiere)
–	Anterofrontal ridge evenly raised and transverse, abruptly joining frontal ridge to form an inverted T-shaped ridge	6
6(5)	Body shiny brownish-black (Fig. 16). Vertex with 4 long and 6 short setae (Fig. 18). Elytra sparsely pubescent (Fig. 16 & 17). Apex of aedeagus narrowing abruptly (Fig. 19). Spermatheca with thin elongate pump (Fig. 21)	*Orthaltica terminalia* sp. n.
–	Body dark brown (Fig. 9). Vertex with 8 long and more than 10 short setae (Fig. 11). Elytra densely pubescent (Fig. 9 & 10). Apex of aedeagus narrowing gradually (Fig. 12). Spermatheca with short thick pump (Fig. 14)	*Orthaltica syzygium* sp. n.

## Discussion

Besides *Orthaltica*, species of *Terminalia* and *Syzygium* also support other leaf feeding beetles belonging to the family Chrysomelidae, as well as to the family Curculionidae. These beetles, being much larger (length 2.2 – 4.5 mm) than *Orthaltica* (length 1.2 – 1.6 mm), feed by cutting holes in the laminae. The adults of *Orthaltica* are extremely small and incapable of cutting holes in the laminae, but rather produce elongate feeding trenches on their adaxial surface. Adults of *Orthaltica syzygium* and *Orthaltica terminalia* utilise holes previously made by larger herbivores, in the leaves of their respective hosts, as shelter. They then create feeding trenches which radiate from the leaf hole which is their shelter (Fig. 1). Beetles could be seen inside holes during most of the day when they were not feeding. They came out of their leaf-hole shelters to feed, forming irregular trenches radiating from the hole. When threatened by means of a finger or a stick, beetles in holes immediately shifted their position to the reverse side of laminae, thus making themselves invisible to the enemy on the opposite side. It was observed that whenever an ant appeared on one side of the lamina, the occupant of a leaf-hole shelter also shifted its position to the reverse side.

Leaf beetles of the subfamily Eumolpinae are the most common primary hole makers on *Syzygium* and *Terminalia* species. At Kudremukha National Park it was observed that a *Basilepta* sp. (Eumolpinae) leaf beetle chewed holes in the laminae of *Syzygium caryophyllatum* (Fig. 3). *Orthaltica syzygium* released on the leaf readily occupied the leaf-hole as a shelter (Fig. 4). By the second day feeding trenches were observed radiating from the leaf-hole shelter (Fig. 5).

The triangular artificial holes, made in the laminae of *Syzygium travancoricum* with a punch, were accepted as shelters (Fig. 6). It was observed that the shape and size of the holes were not exactly what the beetles required. Holes were then resized by partitioning—walls were constructed using faecal pellets (Fig. 7). A single occupant was observed in a hole in most cases. However, when high population densities occurred, several beetles could be seen in a single hole that was large enough to accommodate them (Figs 1, 8).

In the case of the triangular artificial leaf-holes, beetles had a distinct preference for the narrow vertex of the triangle, above its wider base (Fig. 7). This may be because their size allowed them to easily fit inside the narrow apical angle. On 20^th^ May it was found that, of the 316 triangular holes examined on 53 leaves after a month, 235 were occupied. Similarly on 20th August, after a period of four months, of the 319 triangular holes on 58 leaves, 240 were occupied.

Even when suitable leaf-holes were available on leaf laminae, non-shelter forming species never occupied such holes (Fig. 2). *Orthaltica syzygium* confined on leaves without holes fed normally on the adaxial surface of the laminae.

Leaf-hole shelters provide a roosting site that offers a certain degree of camouflage as well as protection. In the field it was observed that on sensing the presence of an enemy on one side of the leaf the occupant of a leaf-hole shelter could easily shift to the other side, making itself invisible to the intruder. It may also be presumed that larger predators cannot pass through the hole in pursuit of the occupant. The leaf-hole, as well as the surrounding area with feeding trenches, turn dark brown and provide a dark background from which the beetle cannot be easily differentiated by a potential predator. On the other hand, the leaf-hole shelter can also provide sufficient cues for a specialist enemy that only needs to learn to locate the leaf-hole shelter to get the inmate. This is the case where insect collectors are concerned—it is easy for them to locate leaf-hole shelter and collect the beetles utilising them.

Leaf-hole shelters were observed in two of the five *Orthaltica* species found in southern India. This is certainly a behavioural novelty of evolutionary significance. It can be seen as an example of Lorenz’s (1937) discovery that a behaviour pattern can be treated as an anatomical organ, which Dawkins (1982) further synthesized and developed into the hypothesis of “the extended phenotype”. It is likely that this extracorporeal adaptation evolved in two lineages of *Orthaltica* in response to interactions with other leaf feeding beetles. Further investigation into the prevalence and diversity of herbivorous beetles on the host plants of *Orthaltica*, and their interactions with each other, might provide the answer.

Adult chrysomelids, unlike their larvae, never carry their faeces in the form of defensive shields. However, females of many species are known to defecate on their eggs after oviposition. Adults are also known to defecate when seized (Müller and Hilker 2004). The use of excreta for the construction of defensive structures or retreats was, until now, not known for adult leaf beetles.

## Supplementary Material

XML Treatment for Orthaltica syzygium

XML Treatment for Orthaltica terminalia
